# Two Cases of Inferior Dislocation of the Patella with Impaction into the Femoral Trochlea of Osteophytes on the Superior Pole of the Patella

**DOI:** 10.1155/2013/691739

**Published:** 2013-12-09

**Authors:** Shinji Yoshioka, Yuji Arai, Kazuya Ikoma, Shinya Fujita, Takanori Akai, Ryuichi Sakuragi, Katsuhito Muneyasu, Toshikazu Kubo

**Affiliations:** ^1^Department of Orthopedic Surgery, Saiseikai Kyoto Hospital, Kyoto, Japan; ^2^Department of Orthopaedics, Graduate School of Medical Science, Kyoto Prefectural University of Medicine, Kyoto 602-8566, Japan

## Abstract

Traumatic dislocation of the patella is classified as lateral, medial, or intra-articular according to the direction of dislocation. Lateral dislocation is the most common type of patellar dislocation, and intra-articular dislocation is rare. Intra-articular dislocation is classified as superior, inferior, or vertical dislocation. Inferior dislocation is categorized as Type I, which occurs in young people, and Type II, which occurs in the elderly. In Type II, osteophytes on the superior pole of the patellar are believed to become entrapped in the intercondylar notch, dislocating the patella inferiorly. These were two extremely rare cases of inferior dislocation of the patella in elderly people. The mechanism involved was considered to be the exertion of sudden upward traction on the patella due to the muscular force of the quadriceps when the knee was flexed, causing osteophytes on the superior pole of the patella to become impacted into the femoral trochlea. Dislocations were successfully reduced without anesthesia, and osteophyte resection or complete osteophyte fracture during reduction meant that there was no recurrence of the dislocation.

## 1. Introduction

Traumatic dislocation of the patella is classified as lateral, medial, or intra-articular according to the direction of dislocation. Lateral dislocation is the most common type of patellar dislocation, and intra-articular dislocation is rare. Intra-articular dislocation is classified as superior, inferior, or vertical dislocation. Inferior dislocation is categorized as Type I, which occurs in young people, and Type II, which occurs in the elderly. In Type II, osteophytes on the superior pole of the patellar are believed to become entrapped in the intercondylar notch, dislocating the patella inferiorly. We treated two extremely rare cases in which osteophytes on the superior pole of the patella became impacted in the femoral trochlea, causing inferior dislocation of the patella.

## 2. Case 1

**Figure 1:**
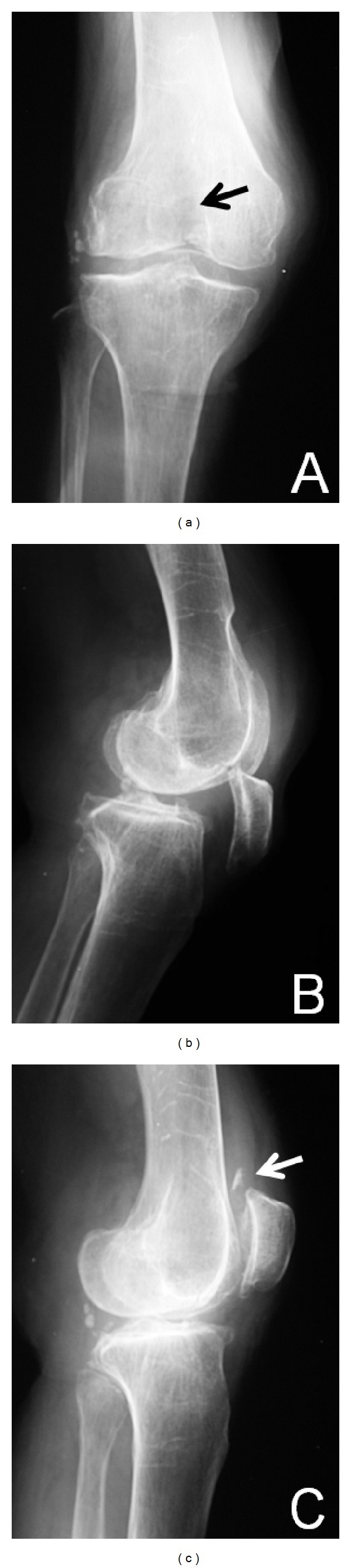
(a) Plain frontal radiography: an intra-articular fracture (arrow) is evident from the condylar fossa to the lateral condyle of the femur. (b) Plain lateral radiography: the patella is dislocated inferiorly. (c) Plain radiography after manual reduction: the remaining osteophyte and a floating bone fragment are visible (arrow).

**Figure 2:**
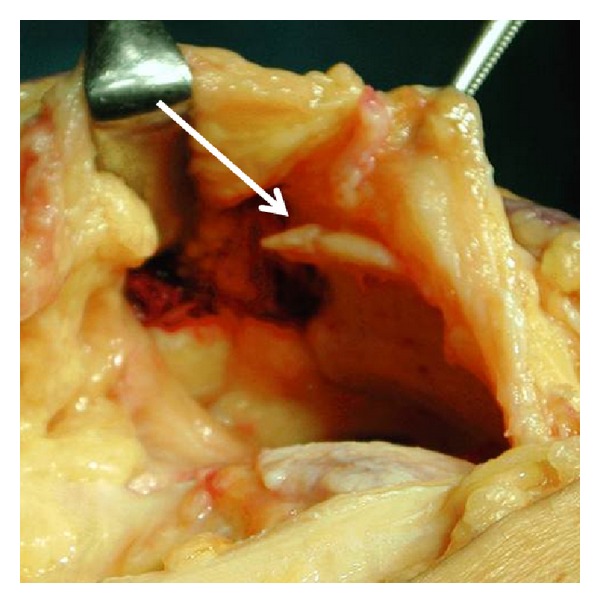
Intraoperative findings. Osteophyte is apparent on the superior pole of the patella (arrow).

A 90-year-old woman presented with pain and locking of the right knee. She was unable to walk after stumbling over a curb and falling. On initial examination, the right knee was locked at an angle of 40° and could not be moved voluntarily. Local findings comprised a depression on the anterior surface of the right knee and a bony protuberance immediately distal to this. The right knee was swollen. No signs of trauma were seen, such as skin abrasions or subcutaneous hemorrhage. In terms of imaging findings, plain frontal radiography of the right knee showed inferior displacement of the patella with a fracture line on the lateral condyle of the femur (Figure 1(a)). Lateral radiography showed that an osteophyte on the superior pole of the patella had become impacted in the femoral trochlea (Figure 1(b)). No rotational abnormality of the patella was evident. The inferiorly dislocated patella was easily reduced when the knee was gently extended and was accompanied by a grating sound. Patellar reduction was confirmed on plain frontal and lateral radiography. Lateral radiography also showed an osteophyte on the superior pole of the right patella with a floating bone fragment nearby (Figure 1(c)). Sagittal magnetic resonance imaging (MRI) showed that the insertion of the femoral quadriceps tendon was not detached and no rupture of the tendon was present. Surgery was later performed with the aim of achieving early ambulation. Intraoperative findings revealed a pointed osteophyte 1.5 cm × 1.5 cm in size on the superior pole of the patella and a floating bone fragment was believed to have resulted from the fracture of the osteophyte (Figure 2). Insall-Salvati ratio for assessment of patella height was 1.07. There was no finding of patella baja. The osteophyte was resected as far as possible, and the bone fragment was removed. Fixation of the fracture of the lateral femoral condyle was carried out using a May anatomical bone plate (KiSCO Corp, Kobe, Japan). The right knee was immobilized in the extended position with a plaster slab for 1 week after surgery, and range of motion training of the right knee was started on postoperative day 7. Partial weight-bearing was initiated from week 5, and full weight-bearing was permitted from week 9. Range of motion at 15 months postoperatively was 0° extension and 120° flexion, and plain radiography showed that synostosis had been achieved. No recurrence of inferior dislocation of the patella has been identified, and the patient has followed an uneventful course and is ambulatory.

## 3. Case 2

**Figure 3:**
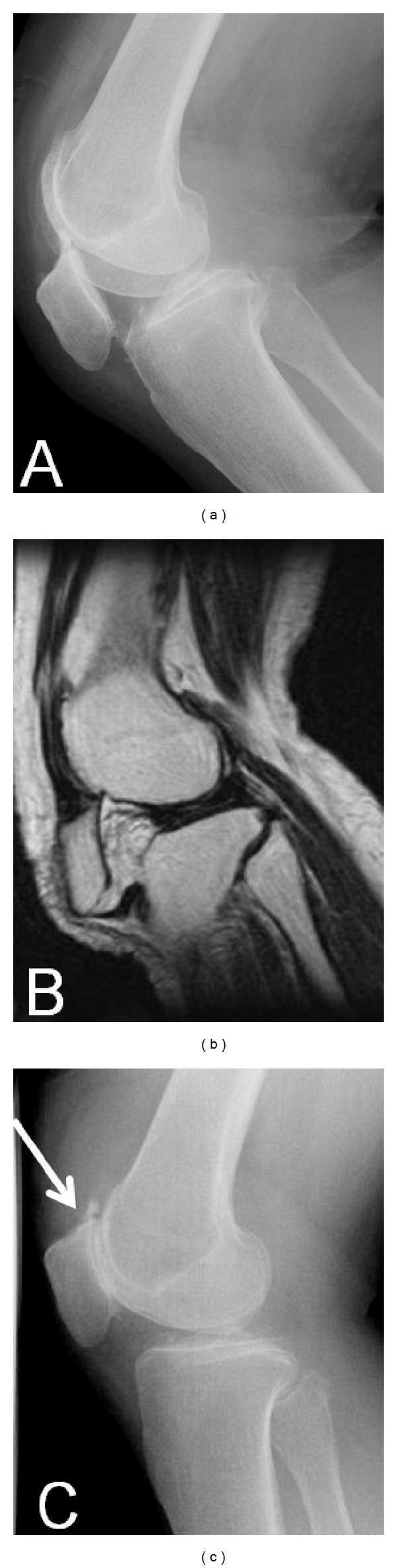
(a) Plain lateral radiography: the patella is dislocated inferiorly. (b) MRI: the osteophyte on the superior pole of the patella has become impacted in the femoral trochlea. (c) Plain radiography after manual reduction: the osteophyte on the superior pole of the patella is completely fractured (arrow).

A 76-year-old woman presented with pain and locking of the right knee. She had attempted to sit on a chair fitted with casters and it had slid away behind her, resulting in her almost falling. She had suddenly extended her knee to avoid falling, at which point it locked and she became unable to move it. On initial examination, the right knee was locked at an angle of 80° and could not be moved voluntarily. Local findings comprised displacement of the right patella to below the knee. There were no signs of trauma such as skin abrasions or subcutaneous hemorrhage. Plain lateral radiography of the right knee showed that an osteophyte on the superior pole of the patella had become impacted in the femoral trochlea and the patella was displaced inferiorly (Figure 3(a)). There was no rotational abnormality of the patella. MRI clearly showed that the osteophyte on the superior pole of the patella was impacted in the femoral trochlea (Figure 3(b)). The insertion of the femoral quadriceps tendon was not detached, and there was no rupture of the tendon. The patient suffered from osteoporosis, with a low bone mineral density of 0.583 g/cm^2^ (*T* score −2.5 SD) prior to the injury. After injection of 0.5% xylocaine 20 mL into the right knee joint, the superior pole of the patella was pressed downward while the knee was gently extended. The right patella was reduced by this procedure, and locking of the right knee was released. Patellar reduction was confirmed on plain lateral radiography (Figure 3(c)). Insall-Salvati ratio was 0.9. There was no finding of patella baja. The osteophyte on the superior pole of the patella was completely fractured, and the choice was made to use conservative treatment as it was believed that recurrent dislocation was unlikely. The right knee was immobilized in the extended position with a plaster slab for 3 weeks after reduction. Range of motion training was started from week 4, and full weight-bearing walking was permitted from week 5. Range of motion at 21 months after injury was 0° extension and 130° flexion, and there has been no recurrence of inferior dislocation of the patella. The patient has followed an uneventful course and is ambulatory.

## 4. Discussion

Bankes and Eastwood classified inferior dislocations of the patella into two types according to the orientation of the articular surface of the dislocated patella and the presence of osteophytes [1]. Type I occurs when a direct blow to the flexed knee forces the superior pole of the patella into the intercondylar notch. This also results in detachment of the insertion of the femoral quadriceps tendon, and the patella is rotated in the horizontal plane so the articular surface faces inferiorly. This commonly occurs in young men. In Type II, unlike Type 1, osteophytes on the superior pole of the patella become entrapped in the intercondylar notch, dislocating it inferiorly, but there is no detachment of the insertion of the femoral quadriceps tendon, and the articular surface is not rotated in the horizontal plane [1]. This type is caused by osteophytes and commonly occurs in elderly women. The two cases reported here were both classified as Type II on the basis of a lack of rotational abnormality of the patella in the horizontal plane and the presence of osteophytes, but the fact that these dislocations were caused by osteophytes on the superior pole of the patella becoming impacted in the femoral trochlea makes them extremely rare.

Two mechanisms for the occurrence of Type II inferior dislocation of the patella have been reported [2]. In one mechanism, a direct upward blow to the inferior pole of the patella from below when the knee is flexed causes osteophytes on the superior pole of the patella to become trapped in the intercondylar notch, resulting in dislocation. In the other, the muscular force of the quadriceps when the knee is flexed exerts upward traction on the patella, causing osteophytes on the superior pole of the patella to become trapped in the intercondylar notch and resulting in dislocation in the absence of a direct blow. In the cases reported here, there were no signs of trauma on the knee such as skin abrasions or subcutaneous hemorrhage. In Case 2, in particular, the dislocation occurred when the patient suddenly tried to extend their knee from a flexed position, suggesting that the latter mechanism was involved. In both patients, however, the bone of the femoral condyle may have been fragile, as Case 1 was very elderly, at 90 years old, and Case 2 had low bone mineral density prior to the injury. This may have been why the osteophytes on the superior pole of the patella became impacted in the weakened femoral trochlea, rather than entrapped in the intercondylar notch when rapid upward traction was applied to the patella, causing dislocation.

Many reports have described the successful closed reduction of this dislocation. Some authors have reported that general anesthesia was required for reduction, [3–5] whereas others have stated that it could be performed without anesthesia [6] or with administration of sedatives alone [1, 2, 7]. Various reduction procedures have been reported, including extending the knee after flexion, [6] applying traction to the tibia to push the inferior pole of the patella upward from below, [7] and pressing on the superior pole of the patella while extending the knee [5]. According to Murakami, [8] whether closed reduction is feasible depends on the degree to which the patella is entrapped in the intercondylar notch. In the cases reported here, plain radiography after reduction confirmed fractures of the osteophytes on the superior poles of the patellas, suggesting that reduction was easily performed without anesthesia because the osteophytes fractured during the reduction procedure.

Bankes and Eastwood [1], Joshi [4], and Garner et al. [7] all reported no recurrence of dislocation after manual reduction alone. Nielsen et al. [9] and Syed and Ramesh [2], however, reported that dislocation recurred and stated that osteophyte resection was necessary to prevent recurrence. Barlow et al. [10] also reported the occurrence of recurrent dislocation in a case of inferior dislocation of the patella in which an osteophyte on the superior pole of the patella had become impacted in the lateral condyle of the femur, with treatment by arthroscopic removal of the osteophyte and trimming of the articular surface of the lateral condyle. In Case 1, open osteosynthesis of the femoral condyle fracture was performed with the aim of early ambulation, during which the remaining osteophyte on the superior pole of the patella was resected and the floating osteophyte removed. In Case 2, conservative treatment was used because the osteophyte on the superior pole of the patella had completely fractured after reduction. As a result, there was no recurrence of inferior dislocation of the patella in either patient. Treatment of osteophytes and monitoring of their condition after reduction are important to prevent recurrence of inferior dislocation of the patella.

The patient in the present case was informed that the data in her case would be submitted for publication.
